# Eye Gaze during Observation of Static Faces in Deaf People

**DOI:** 10.1371/journal.pone.0016919

**Published:** 2011-02-16

**Authors:** Katsumi Watanabe, Tetsuya Matsuda, Tomoyuki Nishioka, Miki Namatame

**Affiliations:** 1 Research Center for Advanced Science and Technology, The University of Tokyo, Tokyo, Japan; 2 Human Technology Research Institute, National Institute of Advanced Industrial Science and Technology, Tsukuba, Japan; 3 Japan Science and Technology Agency, Saitama, Japan; 4 Brain Science Institute, Tamagawa University, Tokyo, Japan; 5 Department of Synthetic Design, Tsukuba University of Technology, Tsukuba, Japan; Kyushu University, Japan

## Abstract

Knowing where people look when viewing faces provides an objective measure into the part of information entering the visual system as well as into the cognitive strategy involved in facial perception. In the present study, we recorded the eye movements of 20 congenitally deaf (10 male and 10 female) and 23 (11 male and 12 female) normal-hearing Japanese participants while they evaluated the emotional valence of static face stimuli. While no difference was found in the evaluation scores, the eye movements during facial observations differed among participant groups. The deaf group looked at the eyes more frequently and for longer duration than the nose whereas the hearing group focused on the nose (or the central region of face) more than the eyes. These results suggest that the strategy employed to extract visual information when viewing static faces may differ between deaf and hearing people.

## Introduction

It has been hypothesized that deaf people may explore and see the visual world differently from hearing people because of their adaptation to hearing loss and/or consequential changes in communication strategy. Some studies have supported altered visual functions in deaf people, especially in the distribution and processes of visual attention [1] [6].

Facial processing is considered to be one of the fundamental visual processes necessary for successful social interaction. This is because, for sighted people, facial processing constitutes a basic skill for detecting and recognizing other people's emotional states. A few studies have shown that facial processing in deaf people might differ from that of hearing people. For example, McCullough & Emmorey [7] showed that American deaf people are better at detecting subtle differences in facial features (particularly around the eyes and mouth) and suggested that long-term experience in discriminating grammatical facial expressions used with American Sign Language (ASL) and lip-reading may contribute to enhanced detection of nuances in relevant facial features (see also [8],[9]).

Since high spatial resolution visual processes are possible only at the fovea, humans produce a series of foveal fixations to extract visual information [10], which are closely linked with overt visual attention [11]. With regard to facial processing, studies investigating eye movements have consistently found a systematic fixation sequence in which the eyes are not directed equally to all regions of a face but only to selected parts; i.e., mainly the eyes and mouth [12] [16].

Several studies that examined the eye movements of deaf people have found that they tend to look at facial regions in a similar magnitude as do hearing people [17] [–] [19]. For example, Muir & Richardson [17] conducted gaze-tracking experiments with deaf people watching sign language video clips and found that participants fixated mostly on the facial regions rather than on the hand movements of the signer, presumably to detect facial movements related to expression. In addition, Emmorey et al. [19] compared eye movements of beginning signers with experienced signers of ASL during ASL comprehension and found differences in fixation patterns: Beginning signers looked at facial regions around the signer's mouth while native signers fixated more on the areas around the eyes. Although these previous studies showed that there are minor differences in fixation patterns between certain groups, the sequence of fixation on the eyes and mouth has been considered to be a universal information extraction pattern.

Nevertheless, the idea of strictly universal facial processing has recently been challenged by several studies that investigated cultural influences on eye movements [20],[21]. Blais et al. [20] showed that Western Caucasian observers consistently fixated on the eye region and partially on the mouth area, confirming the triangular fixation pattern, whereas East Asian observers fixated more on the central region of the face (i.e., around the nose region). These results were interpreted by the authors in the context of cultural influences on visual environment affordance (analytic versus holistic processes [22]) and indicate that, even for face processing, strategies employed to extract visual information are shaped by experience (see also [23] [27]).

Since hearing loss imposes significant constraints on everyday life, it is possible that deaf people use a visual strategy that is different from that used by hearing people, which might lead to differences in scan paths. The triangular pattern of scan path during observations of faces has not been examined quantitatively in deaf people. The purpose of the current study was therefore to report differential scan paths between deaf and hearing people. We chose an emotional valence evaluation task with static faces because it was easy to understand and perform by both deaf and hearing participants.

Because our participants were Japanese (i.e., East Asian), we expected to observe the generally dominant fixations on the central region of the face (i.e., around the nose region) [20]. Then, there were several possibilities, besides that of no difference in eye movement pattern between deaf and hearing participants. Firstly, since deaf people communicate with sign languages, manually signed languages, and/or lip-reading, the mouth region would be of importance for deaf people and therefore fixated more during face observation. Secondly, eye contact is an imperative component of communication and this is more so in a deaf community [28]. Hence, fixations in the eye region might be more pronounced in deaf people. Thirdly, visual processing in deaf individuals exhibit more emphasis on the peripheral visual field [2] [6]. Therefore, in addition to the general tendency toward the nose region [20] [21], deaf individuals might make more eye movements in the parafoveal and peripheral regions, irrespective of whether the region is the main parts of faces (eyes and mouth) or not.

## Methods

### Ethics Statement

The procedures were approved by the internal review board of the Tsukuba University of Technology, and written informed consent was obtained from all participants prior to the testing.

### Participants

We recruited 24 congenitally deaf Japanese people and 29 Japanese people with normal hearing function. Due to procedural failures during the experiment and/or spontaneous withdrawal from the study, data from 10 participants were excluded. The remaining participants comprised 20 congenitally deaf Japanese people (10 males and 10 females; mean age  = 21.7 years, standard deviation  = 0.75) and 23 Japanese people with normal hearing function (11 males and 12 females; mean age  = 24.6 years, standard deviation  = 3.11). All deaf participants were undergraduate students at Tsukuba University of Technology, where one of the entrance criteria is hearing loss of 60 dB or more. The deaf participants typically used manually signed Japanese and/or lip-reading for communication. None of the hearing participants were practiced in sign languages, manually signed languages, or lip-reading.

### Stimuli

Stimuli were obtained from a commercially available database (the ATR face database DV99; ATR-Promotions, Inc.) and consisted of 4 male and 4 female Japanese identities expressing 10 different expressions (neutral [NE], fear [FE], happiness with the mouth opened [HO], happiness with the mouth closed [HC], sadness [SD], surprise [SP], anger with the mouth opened [AO], anger with the mouth closed [AC], disgust [DI], and contempt [CT]). The images were displayed on a 17-inch LCD monitor and viewed at a distance of about 55 cm, subtending 20 degrees of visual angle (27 cm) vertically and 36 degrees of visual angle (54 cm) horizontally. Each face image was centrally located and about 20 cm in height, which represents the size of a real face. Approximate positions of the eyes and mouth were aligned. Presentation of stimuli was controlled by Tobii Studio software (ver. 2.1.12, Tobii Technology, Stockholm, Sweden).

### Eye tracking

Eye movements were recorded at a sampling rate of 60 Hz with the Tobii T-60 eye-tracker (Tobii Technology), which has an average gaze position error of 0.5 degrees and near-linear output over the range of the monitor used. Only the dominant eye of each participant was tracked although viewing was binocular. A manual calibration of eye fixations was conducted at the beginning of each session using a 9-point fixation procedure as implemented in the Tobii Studio software, and drift correction was performed for each trial.

### Procedure

Participants were informed that they would be presented with a series of face pictures in order to evaluate the emotional valence of each face stimulus shown. Before each trial, participants were instructed to fixate on a cross at the center of the screen to perform an automatic drift correction. The participant initiated each trial by pressing a space bar. After a 2-s fixation period, a face was presented for 3 s. Then, the evaluation display appeared, and the participants used a computer mouse to click on the emotional valence of the face in the picture (out of a 7-point positive-negative scale with 1 being most positive). Evaluation was not speeded. Upon the participant's click of the mouse, the next trial began. A session consisted of 3 training trials with neutral expressions followed by 144 test trials. For the test trials, each combination of 8 identities and 9 expressions (except for NE) was presented once (72 trials), and each neutral expression of 8 identities was repeated 9 times (72 trials). The presentation of face stimuli was randomized.

### Data analysis

The rating scores of emotional valence were first averaged for each expression by each participant. The mean rating scores were then grouped by the combination of hearing loss and participants' gender. A 3-way analysis of variance (ANOVA) was conducted to assess statistical significance, with hearing loss (deaf versus hearing) and participants' gender (male versus female) as between-group factors and facial expression of the stimulus as a within-group factor.

**Figure 1:**
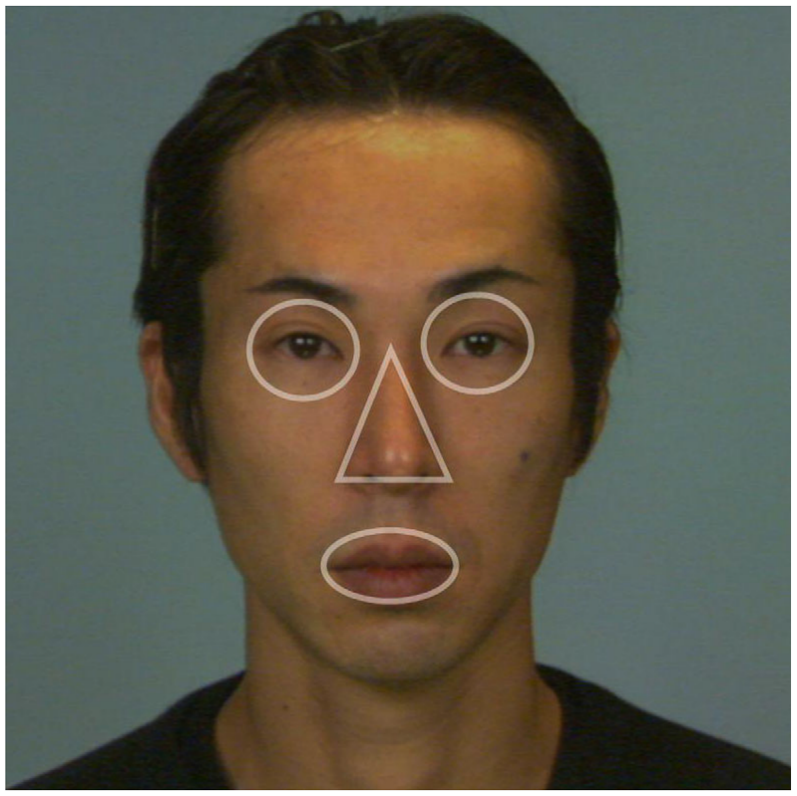
Example of areas of interest (AOIs). For each face stimulus, we defined AOIs: the eyes, nose, and mouth. In order to control for differences in sizes of AOIs, the fixation duration and fixation frequency were normalized by the AOI (relative fixation duration and relative fixation frequency) so that the sum of fixation duration and that of fixation frequency would be 1 for each trial.

For each participant, we calculated the time that they fixated (fixation duration) and the number of fixations (fixation frequency) on the following areas of interests (AOIs): the eyes, the nose, and the mouth. AOIs were defined for each face (Figure 1). To control for differences in the sizes of AOIs, we normalized the fixation duration by the area of the AOI so that the sum of relative fixation duration would be 1 for each trial (relative fixation duration). The same normalization was performed for fixation frequency to calculate relative fixation frequency. Relative fixation duration and relative fixation frequency on the different AOIs were averaged separately for expressions within each participant. The averages were then grouped by combining hearing loss and participants' gender separately for AOIs. The relative fixation duration on AOIs was entered into a 4-way ANOVA, with hearing loss and participants' gender as between-group factors and facial expression and AOIs as within-group factors. The same ANOVA was conducted on the relative fixation frequency.

## Results

### Evaluation of emotional valence

**Figure 2:**
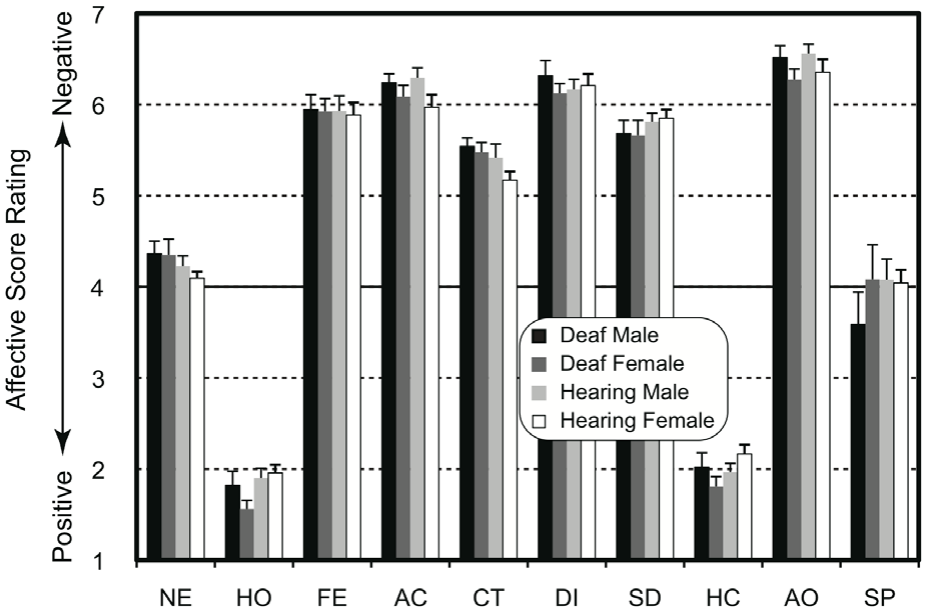
Mean rating scores of emotional valence as a function of expression in face stimuli. The face stimuli with happy expressions (HO and HC) were evaluated positively while the face stimuli with sad (SD), angry (AO and AC), disgust (DI), and contempt (CT) expressions were rated negatively. The faces with neutral (NE) and surprised (SP) expressions were evaluated neither positively nor negatively. NE =  neutral; HO =  happiness with the mouth opened; FE =  fear, AC =  anger with the mouth closed; CT =  contempt; DI =  disgust; SD =  sadness; HC =  happiness with the mouth closed; AO =  anger with the mouth opened; and SP =  surprise.

The averaged rating scores of emotional valence are shown in Figure 2. Face stimuli with happy expressions (HO and HC) were evaluated positively while face stimuli with fear (FE), sad (SD), angry (AO and AC), disgust (DI), and contempt (CT) expressions tended to be rated negatively. Faces with neutral (NE) and surprised (SP) expressions were evaluated, on average, neither positively nor negatively. Three-way ANOVA showed that a main effect of expression (F(9,351) = 597.7, *P*<0.001) was significant while the main effects of hearing loss and participants' gender were not significant (F(1,39 = 0.3, *P* = 0.60, F(1,39) = 1.2, *P* = 0.29, respectively). No interaction reached a significant level (F<1.3, *P*>0.23). These results suggest that the participants evaluated the emotional valence of the faces presented as stimuli consistently, irrespective of hearing loss and participants' gender.

### Eye movements

**Figure 3:**
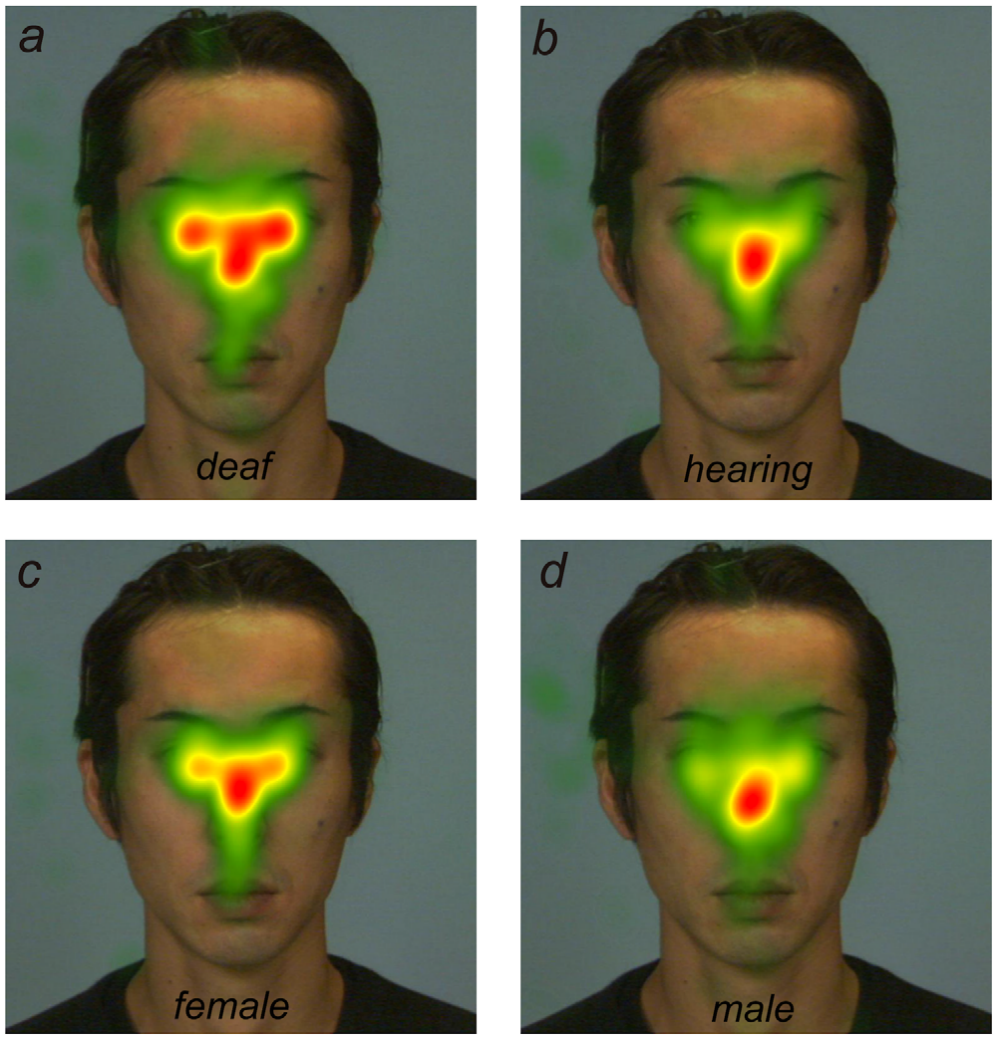
Total fixation duration mapped onto an example face image: (a) deaf participants, (b) hearing participants, (c) female participants, and (d) male participants. Red regions represent the places where the participants' eyes stayed longer. The fixation patters differed among the participant groups.

Data from trials where no gazes were directed at AOIs (i.e., the eyes, nose, or mouth) were excluded from the following analysis, which were 0.8%, 0.4%, 0.4%, and 0.5%, for deaf male, deaf female, hearing male, and hearing female groups, respectively. There was no significant difference in the number of discarded trials (Fisher exact test; *P* = 0.32). Figure 3 depicts the relative fixation duration mapped onto an example image of neutral face separately summed for deaf participants (Figure 3a), normal-hearing participants (Figure 3b), female participants (Figure 3c), and male participants (Figure 3d). This figure suggests that the gaze patterns differed among the participant groups.

**Figure 4:**
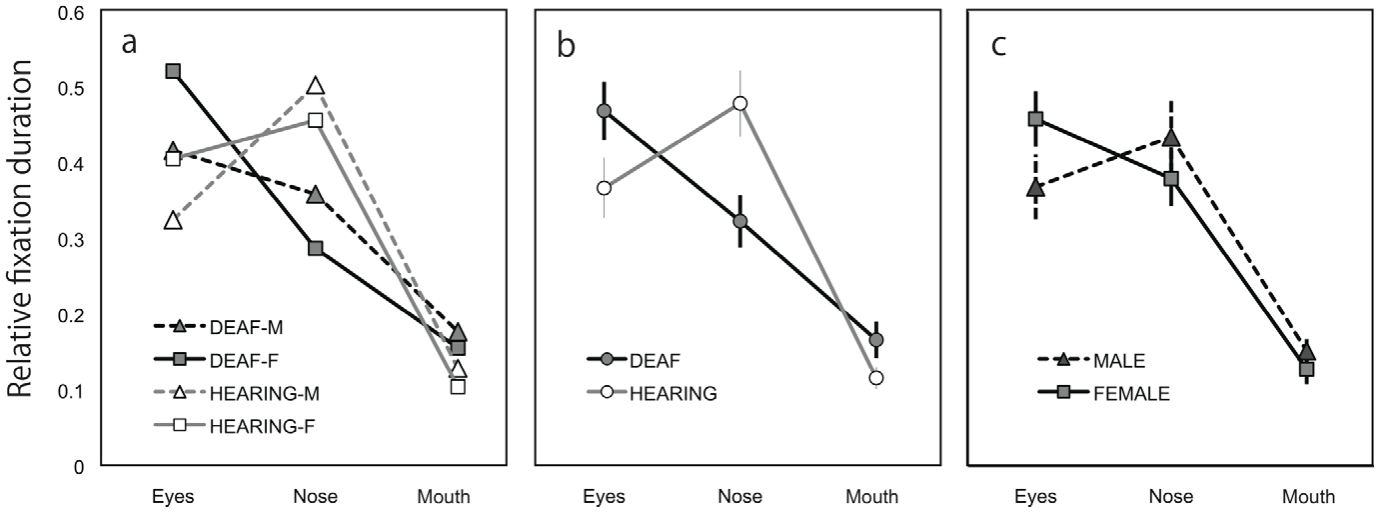
Relative fixation duration. (**a**) Relative fixation duration as a function of area of interest averaged over all facial expressions within different combinations of participant groups. Participants tended to fixate on the eyes and nose longer than on the mouth. (**b**) Relative fixation duration compared between deaf and hearing participants. The deaf participants looked at the eyes longer than the nose whereas the hearing participants gazed at the nose longer than the eyes. (**c**) Relative fixation duration compared between female and male participants. The female participants tended to fixate on the eyes longer than did the male participants.

**Figure 5:**
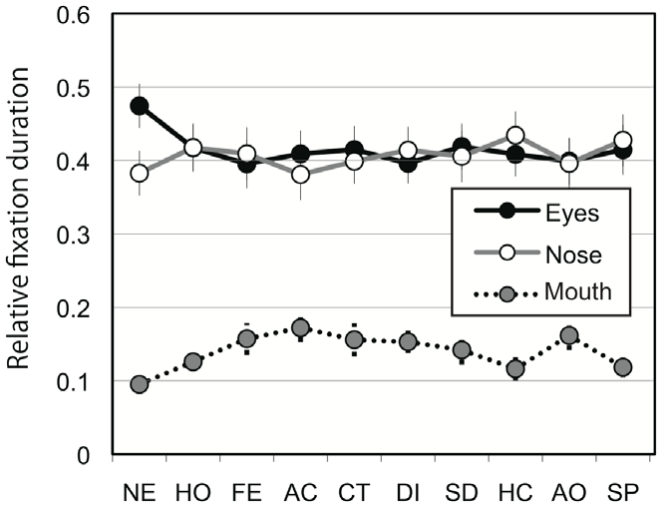
Relative fixation duration for the different areas of interest as a function of facial expression, averaged over all the participants. The faces with neutral expression led to longer fixation duration on the eyes. NE =  neutral; HO =  happiness with the mouth opened; FE =  fear, AC =  anger with the mouth closed; CT =  contempt; DI =  disgust; SD =  sadness; HC =  happiness with the mouth closed; AO =  anger with the mouth opened; and SP =  surprise.

Figure 4a shows relative fixation duration as a function of AOI, averaged over all facial expressions within different combinations of participant groups. In general, participants tended to fixate on the eyes and nose longer than on the mouth. In addition, deaf participants looked at the eyes longer than the nose whereas normal-hearing participants gazed at the nose longer than the eyes (Figure 4b). The tendency to fixate longer on the eyes appeared to be stronger in females compared with male participants (Figure 4c). Figure 5 shows the relative fixation duration for the different AOIs as a function of facial expression averaged over all participants. The differential relative fixation durations for different AOIs were apparent; i.e., fixation duration on the eyes and the nose was longer than on the mouth. In addition, the pictures of faces with neutral expressions appeared to lead to longer fixation on the eyes in exchange for shorter fixation duration on the mouth.

**Figure 6:**
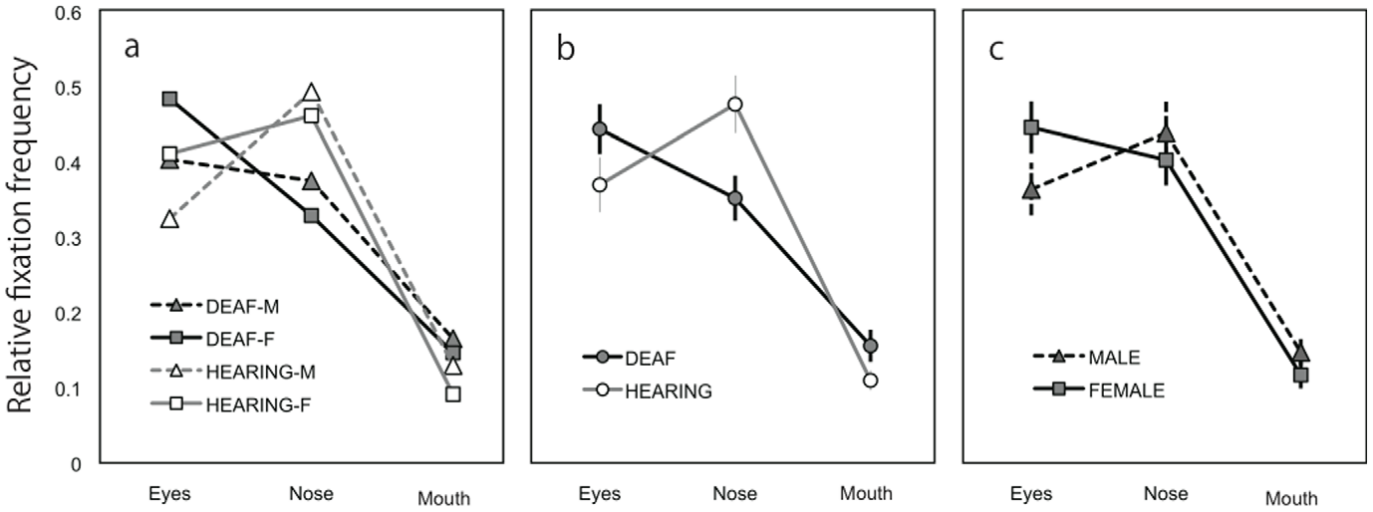
Relative fixation frequency. (**a**) Relative fixation frequency as a function of area of interest averaged over all facial expressions within different combinations of participant groups. (**b**) Relative fixation frequency compared between deaf and hearing participants. (**c**) Relative fixation frequency compared between female and male participants. The results for fixation frequency corroborated those of fixation duration.

**Figure 7:**
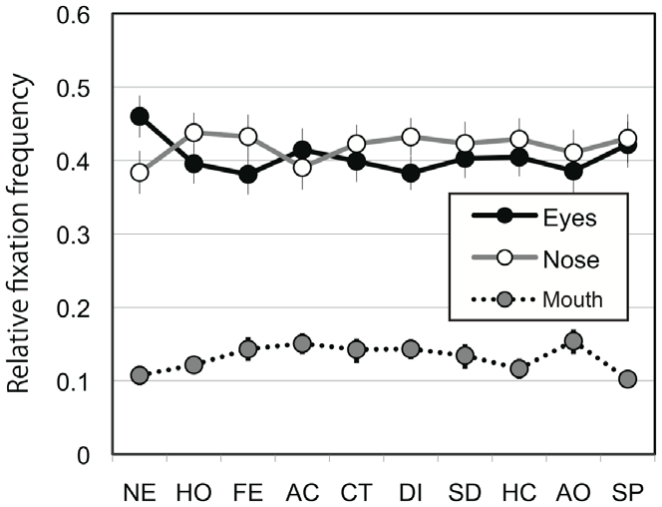
Relative fixation frequency for the different areas of interest as a function of facial expression averaged over all the participants. The results for fixation frequency corroborated those of fixation duration. NE =  neutral; HO =  happiness with the mouth opened; FE =  fear, AC =  anger with the mouth closed; CT =  contempt; DI =  disgust; SD =  sadness; HC =  happiness with the mouth closed; AO =  anger with the mouth opened; and SP =  surprise.

Four-way ANOVA revealed significant main effects of participants' gender (F(1,39) = 8.7, *P*<0.01; female > male), AOI (F(2,78) = 26.8, *P*<0.001; post hoc Ryan's method, eyes  =  nose > mouth, *P*<0.001), and expression (F(9,351) = 5.6, *P*<0.001). A significant interaction between hearing loss and AOI (F(2,78) = 5.3, *P*<0.01) and between expression and AOI (F(18,702) = 2.8, *P*<0.001) were found. There were also significant interactions between hearing loss and expression (F(9, 351) = 2.0, *P*<0.05) and among participants' gender, expression, and AOI (F(18, 702) = 1.7, *P*<0.05). Analyses of simple main effect indicated that the normal-hearing group looked at the nose longer than the eyes whereas the deaf group tended to look at the eyes more than the nose (*P*<0.05). The interaction between expression and AOI was mainly due to the fact that participants fixated longer on the eyes in pictures of neutral faces than in pictures of faces with other expressions (*P*<0.05). The data of relative fixation frequency corroborated the results of relative fixation duration (Figures 6 and 7).

An additional analysis was performed to test whether the fixation duration and fixation frequency outside the AOIs differ among participant groups. Whereas significant main effects of participants' gender were found (male > female; fixation duration, F(1,39) = 13.6, *P*<0.01; fixation frequency, F(1,39) = 15.1, *P*<0.01), no statistical difference was observed between deaf and hearing participants (fixation duration, F(1,39) = 0.14, *P* = 0.7; fixation frequency, F(1,39) = 1.14, *P* = 0.29), corroborating the results of 4-way ANOVA.

## Discussion

In the present study we examined the possible difference in the pattern of eye movements between congenitally deaf and normal-hearing Japanese individuals while they evaluated the emotional valence of static faces. The results can be summarized as follows: (1) The emotional valence of face stimuli were evaluated consistently irrespective of hearing loss and participants' gender; (2) participants fixated (in terms of frequently and duration) on the eyes and nose more than on the mouth (the main effect of AOI), confirming overall fixation dominance on the eyes; (3) female participants tended to look at the main facial parts (i.e., the eyes, nose, and mouth) more than did male participants (the main effect of participant's gender); (4) faces with neutral expressions induced fixations on the eyes more than did faces with other expressions (the interaction between expression and AOI); and (5) deaf participants looked at the eyes more than the nose whereas normal-hearing participants tended to look more at the nose (the interaction between hearing loss and AOI).

It has been reported that females have an advantage in decoding nonverbal emotion [29] [35] and that females look more at the main parts of the face than do males, with particular emphasis on the eyes [34],[35]. The main effects of participant's gender supported this notion. Although the interaction between participants' gender and AOI and the interaction among participants' gender, hearing loss, and AOI did not reach a significant level, our data clearly showed a tendency in the female participants to fixate on the eyes (Figure 4c and Figure 6c). Thus, the present results may be taken as evidence supporting a gender difference in fixation pattern for faces with emotional expressions [34],[35].

Irrespective of participant group, faces with neutral expressions tended to produce more fixations on the eyes than did faces showing other expressions. Faces with emotional expressions have distinct features that help observers to interpret the expression. On the other hand, neutral faces are ambiguous and lack the visual cues for comprehension of emotion. It has been suggested that understanding and communication of emotion depends greatly on the visual processing of the eye region [36] [39]. Therefore, it is possible that people fixate more on the eyes of ambiguous neutral faces in an attempt to discern emotional clues. However, it should be stated that there was a possible confound in the present experiment that the neutral faces were repeated 9 times while the others were presented once and the interaction between expression and AOI may be due to repetition rather than expression. Further investigations are warranted to examine whether less emotional facial expressions indeed lead to more fixation on the eye region. Specifically, a future study should avoid the possible confound between viewing less emotional face expressions and repeated viewing.

The main focus of the present study was to investigate potential differences in fixation patterns between deaf and hearing participants. The hearing participants in the present study looked at the nose (i.e., central) region most rather than at the eye region. Since all the participants in the current study were Japanese, this may be attributed to a cultural influence on eye movement. Blais et al. [20] reported that Western Caucasian observers consistently fixated the eye region, and partially the mouth, whereas East Asian observers fixated more on the central region of the face to extract information from faces. They hypothesized that this difference is due to the social norm in East Asian cultures that direct or excessive eye contact may be considered rude [40] and to the difference in cognitive strategy (holistic/analytic approach to visual information: [22],[41]). On the other hand, our Japanese deaf participants looked at the eye region most, closer to the fixation pattern of Western Caucasians in Blais et al [20]. It has been reported that in a deaf community, eye contact is vital for communication because avoiding eye contact disrupts communication more profoundly than it does in sighted communities [28]; this holds true for a Japanese deaf community. Therefore, it is possible that the increased fixation on the eye region in our Japanese deaf participants may reflect their communication strategy. In this sense, the present study may be taken as an extension of Blais et al. [20], showing that living in a specific community (more specifically, deaf community in Tsukuba University of Technology in Japan) might alter how we look at faces (also see [23] [27]).

The underling mechanism for differential scan paths between deaf and hearing individuals remains to be clarified. However, one possible mechanism is the altered distribution and processes of visual attention [2] [5]. Deaf individuals are more distracted by visual information in the parafovea and periphery [5]. Since there was no difference in fixation duration and frequency outside the AOIs and no increase of fixation in the mouth region, the present finding cannot be explained solely by the attention emphasis on the peripheral processing. However, it is still possible that altered peripheral visual attention and scrutinizing strategy for faces may interact to produce the differential scan paths.

### Limitations of the present study

Although the difference in fixation pattern was clear, it should be noted that the present study has considerable limitations. One limitation is that the stimuli used in the present study were static, rather than dynamic, stimuli. Many studies of emotional expression have used static face stimuli. Yet, facial expressions are highly dynamic, and thus, static stimuli represent unnatural snapshots of them. Recent studies on dynamic facial expressions have shown that visual processes for facial expressions are essentially tuned to dynamic information [36],[42],[43]. Evidence supporting this notion comes from facilitative effects of dynamic presentation on facial processing [44] [50] and enhanced neural activities for dynamic, as opposed to static, face stimuli [51] [–] [53]. Therefore, it is likely that the pattern of results would be different if dynamic stimuli were used. In particular, the relatively less fixations in the mouth region might be due to the use of the static face stimuli. It has been shown that the mouth region conveys useful information for emotion discrimination [54] [58], and this seems to be more so with dynamic face stimuli, e.g., [59].

Another limitation stems from the use of the evaluation task of emotional valence. Many previous studies have examined the scan paths during emotion discrimination and identification (e.g., [56],[57]) but little study has employed an evaluation task of emotional valence. Therefore, the present results may not be compared directly with those of the previous studies. Also, in order to elucidate the mechanism for valence evaluation and emotional processes, it is important to consider the relation between the time-course of evaluation processes and eye movement. The face stimuli used in the present study included some variations in visual information for emotional valence evaluation, which in turn would lead to different demands for different face stimuli. Since the decision was not timed, we did not know when the participants reached their decisions. Therefore, the eye movement pattern may reflect either pre-decision or post-decision processes or both.

The final limitation is the demographic peculiarity of the participants. It is possible that the use of sign language (Japanese Sign Language; JSL) leads to enhanced attention to the eye region because changes in eye configurations convey various syntactic distinctions and grammatical information in JSL as in ASL [60],[61]. However, until around 2002, most Japanese schools for the deaf emphasized oral education; i.e., teaching through lip-reading. Although manually signed Japanese (which is a signed form of the Japanese language) has recently started to be used in schools for the deaf, even now Japanese sign language is not officially taught. Therefore, it is difficult to infer whether the difference in fixation pattern is due to the hearing loss itself, to the extended use of sign language, and/or to the specific historical situation of Japanese deaf education.

Despite the above limitations, the present study showed the differential scan paths during observation of static face stimuli between deaf and hearing participants. Further investigations, preferably with speeded response or confidence/difficulty rating of decision, with dynamic stimuli, and with cross-cultural comparisons, will shed light on how and to what extent hearing loss influences how we look at faces and interpret others.
